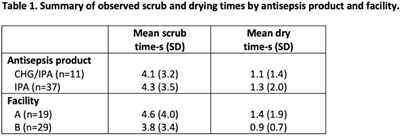# Human factors analysis of the disinfection of central-line needleless connectors

**DOI:** 10.1017/ash.2022.117

**Published:** 2022-05-16

**Authors:** Richard Martinello, Joan Hebden, Frank Drews, David Pegues

## Abstract

**Background:** Patients requiring vascular catheters are at risk for bloodstream infections (BSIs), particularly those with central venous access devices (CVADs). Central-line–associated bloodstream infections (CLABSIs) may occur as a result of the introduction of pathogenic microbes during CVAD access procedures, including through the needleless connector. The use of an antiseptic scrub is recommended to disinfect the needleless connector before device access, and this procedure has been shown to reduce the risk for CLABSI. We identified perceived barriers and facilitators and assessed compliance with instructions for use of chlorhexidine or alcohol antisepsis products (CHG or IPA; 5-second scrub time plus 5-second dry time) and alcohol antisepsis products (IPA; facility protocol 15-second scrub time plus let dry) for needleless connector disinfection. **Methods:** We performed a multiple-methods study involving focus groups composed of a convenience sample of nurses and clinical observations of CVAD needleless-connector access procedures in 3 medical ICUs and 1 surgical ICU at 2 academic medical centers. We used open-ended questions to guide the focus-group discussions. We directly observed nursing staff performing needleless-connector disinfection following a time–motion paradigm using an electronic tool to document the observed needleless-connector access events and to measure needleless-connector antiseptic scrub times and dry times. **Results:** In total, 8 focus groups involving 28 nurses revealed access to the antiseptic product and lesser workload as best-practice facilitators of needleless-connector disinfection. Identified barriers were often the opposite of the facilitators, particularly the time required per needleless connector access using IPA and knowledge deficits regarding the need for disinfection between multiple needleless-connector accesses. From 36 observations, including a total of 48 access events, we determined that the mean scrub times were below the recommended times, especially for IPA (Table 1). Drying time after use of either antisepsis product was negligible. **Conclusions:** A lack of access to the disinfection product, emergency situations, and increased workload were perceived barriers to needleless-connector disinfection. Observed scrub times and drying times were shorter than recommended, much more so for IPA. These deficits in the performance of needleless-connector disinfection may increase the risk of CLABSI. Ongoing education and periodic competency evaluation of needleless-connector disinfection are needed to imbed and sustain best practices.

**Funding:** Professional Disposables, Inc.

**Disclosures:** None

**Table:**